# Inhibition of cytosolic Phospholipase A_2_ prevents prion peptide-induced neuronal damage and co-localisation with Beta III Tubulin

**DOI:** 10.1186/1471-2202-13-106

**Published:** 2012-08-28

**Authors:** Victoria Last, Alun Williams, Dirk Werling

**Affiliations:** 1 Department of Pathology and Infectious Diseases, Royal Veterinary College, Hawkshead Lane, North Mymms, Hertfordshire AL9 7TA, UK; 2 Present address: Department of Hematology, Cancer Institute, University College Medical School, London NW3 2PF, UK; 3 Present address: Department of Veterinary Medicine, University of Cambridge, Madingley Road, Cambridge CB3 0ES, UK

**Keywords:** Prions, Neurotoxicity, Phospholipase A_2_, Synaptophysin, PACOCF_3_

## Abstract

**Background:**

Activation of phospholipase A_2_ (PLA_2_) and the subsequent metabolism of arachidonic acid (AA) to prostaglandins have been shown to play an important role in neuronal death in neurodegenerative disease. Here we report the effects of the prion peptide fragment HuPrP106-126 on the PLA_2_ cascade in primary cortical neurons and translocation of cPLA_2_ to neurites.

**Results:**

Exposure of primary cortical neurons to HuPrP106-126 increased the levels of phosphorylated cPLA_2_ and caused phosphorylated cPLA_2_ to relocate from the cell body to the cellular neurite in a PrP-dependent manner, a previously unreported observation. HuPrP106-126 also induced significant AA release, an indicator of cPLA_2_ activation; this preceded synapse damage and subsequent cellular death. The novel translocation of p-cPLA_2_ postulated the potential for exposure to HuPrP106-126 to result in a re-arrangement of the cellular cytoskeleton. However p-cPLA_2_ did not colocalise significantly with F-actin, intermediate filaments, or microtubule-associated proteins. Conversely, p-cPLA_2_ did significantly colocalise with the cytoskeletal protein beta III tubulin. Pre-treatment with the PLA_2_ inhibitor, palmitoyl trifluoromethyl ketone (PACOCF_3_) reduced cPLA_2_ activation, AA release and damage to the neuronal synapse. Furthermore, PACOCF_3_ reduced expression of p-cPLA_2_ in neurites and inhibited colocalisation with beta III tubulin, resulting in protection against PrP-induced cell death.

**Conclusions:**

Collectively, these findings suggest that cPLA_2_ plays a vital role in the action of HuPrP106-126 and that the colocalisation of p-cPLA_2_ with beta III tubulin could be central to the progress of neurodegeneration caused by prion peptides. Further work is needed to define exactly how PLA_2_ inhibitors protect neurons from peptide-induced toxicity and how this relates to intracellular structural changes occurring in neurodegeneration.

## Background

Prion diseases or transmissible spongiform encephalopathies (TSEs), are fatal neurodegenerative disorders that include bovine spongiform encephalopathy (BSE) in cattle and Creutzfeldt-Jakob disease (CJD) in man. A key event in the pathogenesis of these diseases is the misfolding of the normal prion protein (PrP^C^), to a modified, pathogenic form, termed PrP^Sc^[1]. PrP^Sc^ is the major, if not only, component of the infectious agent of TSEs and PrP^Sc^ itself, or defined fragments of PrP^Sc^, including the synthetic peptide HuPrP106-126 have been demonstrated to be cytotoxic to many different cell types [2-5]. However the exact pathways by which HuPrP106-126 toxicity is mediated remain to be fully elucidated. HuPrP106-126 has the ability to aggregate *in vitro* to form oligomeric fibrils that are insoluble, protease resistant and can aggregate further to form amyloid aggregates [2,6,7]. Effects of HuPrP106-126 on cells include aggregation of PrP^C^ in neuroblastoma cells [7], copper uptake inhibition in cerebellar neurons [8], p38 MAPK activation in correlation with cell death in SH-SY5Y cells [9] and an increase in intracellular Ca^2+^ coupled with membrane viscosity in leucocytes [10].

Previous reports have indicated that the PLA_2_ signalling pathway is implicated in prion disease pathogenesis [11-13]. PLA_2_ isoforms have a predominant role in the central nervous system where they are involved in the pro-inflammatory response, membrane repair, trafficking, neurotransmitter release and apoptosis [14,15]. The main role of the mammalian PLA_2_ enzymes is the production of lipid mediators vital to activate signal transduction and inflammatory pathways more specifically they catalyse the production of free fatty acids from the *sn-2* position in membrane phospholipids via hydrolysis leaving lysophospholipids as a remainder [16]. PLA_2_ enzymes can be divided into three main groups: extracellular, secretory PLA_2_ (sPLA_2_) enzymes, cytosolic PLA_2_ (cPLA_2_) that require smaller amounts (nM) of Ca^2+^ for membrane translocation and calcium-independent PLA_2_ (iPLA_2_) [14,15,17,18]. cPLA_2_ does not require Ca^2+^ to be catalytically active [19], however it does require the presence of nucleophilic Ser-228. In addition, phosphorylation by MAPK, ERK, PKC and CamKII at the conserved residues Ser505, Ser757 or Ser515 can also increase activity [19-21].

The importance of PLA_2_ in the pathogenesis of the neuronal degeneration in prion diseases has been indicated by the use of PLA_2_ inhibitors that reduced PrP^Sc^ formation caspase-3 activity and prostaglandin E_2_ production [11,13,22]. In the present study the effects of the PrP fragment corresponding to amino acid residues 106–126 of human PrP, HuPrP106-126, on the activation of cPLA_2_ and other components of the PLA_2_ pathway was investigated. As cPLA_2_ has been reported to relocate to the nuclear envelope upon activation by the Ca^2+^ ionophore A23187 in CHO cell lines [23], the hypothesis that exposure to HuPrP106-126 leads to changes in cPLA_2_ distribution within neurons was assessed. In addition, a previously untested PLA_2_ inhibitor, palmitoyl trifluoromethyl ketone (PACOCF_3_) was investigated for its effects on HuPrP106-126-induced cPLA_2_ activation and neuronal degeneration. This PLA_2_ inhibitor prevented translocation of PLA_2_ and subsequent synapse degeneration and neuronal death.

In the present study we provide important new insights into the position of cPLA_2_ in the mechanism underlying PrP neurotoxicity and implicate the involvement of the cytoskeleton in prion disease pathogenesis.

## Results

### Exposure of primary cortical neurons to HuPrP106-126 activates cPLA_2_ and induces a novel relocation

The human prion protein peptide fragment HuPrP106-126 was used to investigate the effect of PrP upon PLA_2_ in primary cortical neurons. It is known that cPLA_2_ is promptly activated within 1 hour by agonists including phorbol 12-myristate 13-acetate (PMA) A23187 and ionomycin [24-26], this was confirmed in murine primary cortical neurons via preliminary experiments (Additional file 1: Figure S1), and therefore neurons were initially treated for 30 minutes. p-cPLA_2_ was visualised by confocal microscopy using an anti-phospho cPLA_2_ antibody against the serine-505 residue.

**Figure 1:**
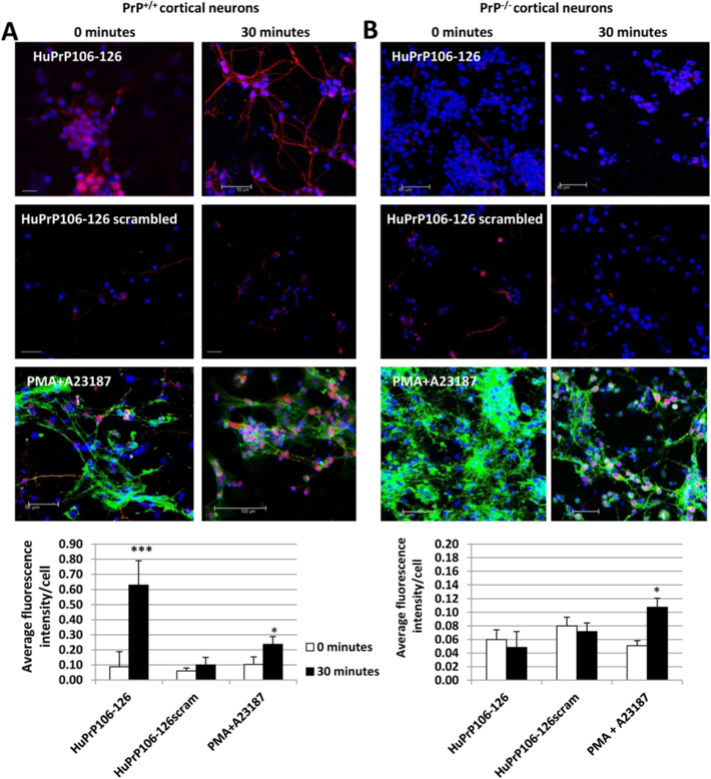
**HuPrP106-126 causes cPLA**_**2 **_**activation and relocation in primary cortical neurons.** (**A**) CD-1 murine primary cortical neurons of an *in vivo* age of 7 days were treated for 0 (left panel) or 30 minutes (right panel) with 40 μM HuPrP106-126 or HuPrP106-126 scrambled peptides, or 1 μM PMA + 5 μM A23187. The cellular localization of p-cPLA_2_ was analyzed by immunofluorescence using anti-p-cPLA_2_ (red) antibody. In addition actin was visualised in PMA + A23187 samples using phalloidin (green). Nuclei were stained with DAPI. Scale bar: 50 μM. Intensity of p-cPLA_2_ labelling was measured and normalised to cell number, shown in accompanying graph. (**B**) PrP null murine primary cortical neurons were treated with 40 μM HuPrP106-126, HuPrP106-126 scrambled or 1 μM PMA + 5 μM A23187. Intensity of p-cPLA_2_ labelling was measured and normalised to cell number. Data expressed as mean ± S.D. of three experiments. *P < 0.05, ***P < 0.001.

In untreated neurons a low basal level of p-cPLA_2_ labelling in the nuclear region could be seen, however exposure to 40 μM HuPrP106-126 resulted in a significant increase in the intensity of p-cPLA_2_ labelling (P < 0.001), indicating amplified levels of the enzyme (Figure 1A). In addition, p-cPLA_2_ appeared to relocate from the cell body to the neurites (Figure 1A), an occurrence not previously noted in cPLA_2_ activation. The effect of HuPrP106-126 on PLA_2_ activation was amino acid sequence specific and dependent on the presence of PrP, as the intensity and localisation of p-cPLA_2_ labelling was not altered in cells exposed to 40 μM scrambled HuPrP106-126 peptide or in PrP null neurons exposed to HuPrP106-126 (Figure 1A and B). In contrast known activators of cPLA_2_, PMA and A23187, induced p-cPLA_2_ activation and localisation to the nuclear region in both cell types (P < 0.05). This is consistent with previous studies which reported that cPLA_2_ translocates to the ER and nuclear membrane upon activation [23].

**Figure 2:**
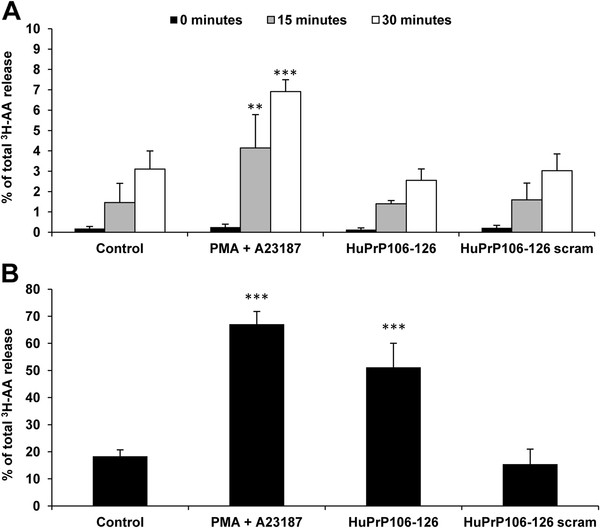
**HuPrP106-126 induces [**^**3**^** H]-AA release after 24 hours in primary cortical neurons.** (**A**) Primary cortical neurons were incubated with 1 μCi ml^-1^ [^3^ H]-AA for 24 hours. Cells were washed twice with media and treated with 1 μM PMA + 5 μM A23187, 40 μM HuPrP106-126 or HuPrP106-126scram for 0, 15 or 30 minutes. Levels of [^3^ H]-AA were measured in cell lysates and supernatant to calculate a% [^3^ H]-AA release. (**B**) Experiment was repeated with cells treated for 24 hours. Data expressed as mean ± S.D. of duplicate samples from three experiments (n = 6). **P < 0.01, ***P < 0.001.

The effect of HuPrP106-126 on the cPLA_2_ cascade was further analysed by measuring the release of arachidonic acid (AA). Cleavage and subsequent release of AA by cPLA_2_ can be used as a marker for PLA_2_ activity. Primary cortical neurons were exposed to 40 μM HuPrP106-126 or scrambled HuPrP106-126. After 15 and 30 minutes PMA and A23187 induced significant [^3^ H]-AA release (P < 0.01 and P < 0.001 respectively) (Figure 2A). In contrast, HuPrP106-126 did not induce [^3^ H]-AA release above untreated or scrambled control levels. When incubation was prolonged to 24 hours, PMA and A23187 again induced a significantly higher [^3^ H]-AA release compared to controls (P < 0.001), and at this time HuPrP106-126 also induced a significant [^3^ H]-AA release (P < 0.001) (Figure 2B). Levels of AA following exposure to scrambled peptide controls remained comparable to untreated controls.

### PACOCF_3_ inhibits HuPrP106-126-induced cPLA_2_ activation and neuronal damage

**Figure 3:**
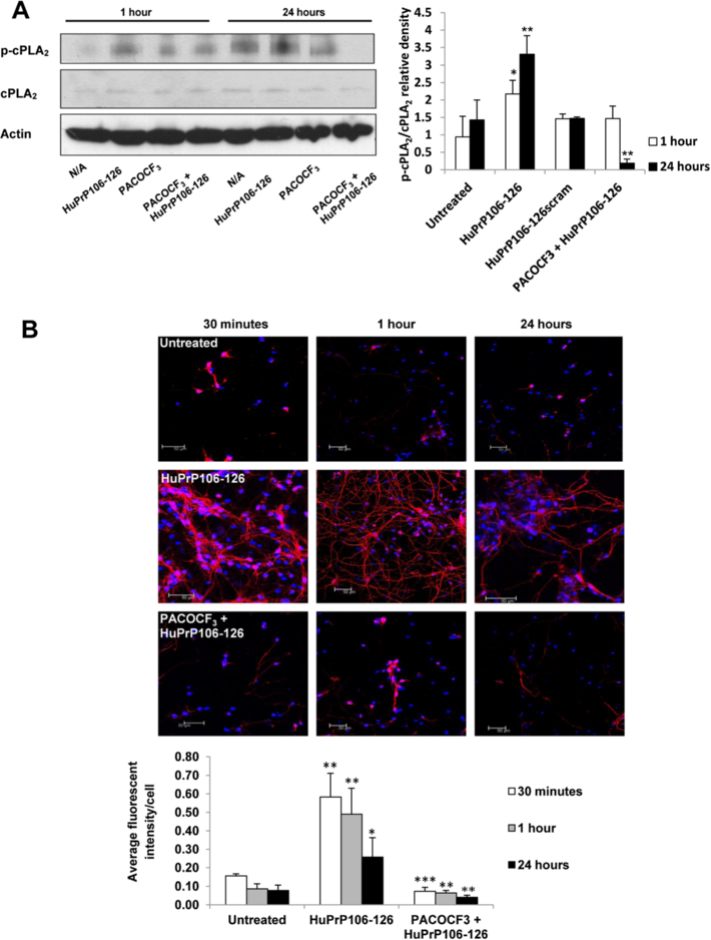
**PACOCF**_**3 **_**prevents HuPrP106-126-induced cPLA**_**2 **_**activation.** (**A**) Primary cortical neurons were treated with 40 μM HuPrP106-126 for 1 hour or 24 hours, or pre-treated with 1 μM PACOCF_3_ before the addition of peptide. Samples were analysed by immunoblotting and the amount of p-cPLA_2_ and total PLA_2_ was calculated by densitometry. (**B**) Neurons were further analysed by confocal microscopy. Samples were treated with HuPrP106-126 for 30 minutes, 1 hour or 24 hours, or pre-treated with PACOCF_3_ prior to HuPrP106-126. p-cPLA_2_ was labelled (red) and nuclei were stained with DAPI. Fluorescence intensity of p-cPLA_2_ was measured and normalised to cell number, shown in the accompanying graph. Data expressed as mean ± S.D. of three experiments *P < 0.05, **P < 0.01, ***P < 0.001.

To examine the specific involvement of cPLA_2_ in HuPrP106-126-induced cellular toxicity cells were pre-treated with the PLA_2_ inhibitor PACOCF_3_, a known cPLA_2_ inhibitor [27]. Primary cortical neuron samples were analysed by Western immunoblotting and levels of activated and total cPLA_2_ were measured. After exposure to 40 μM HuPrP106-126 for a period of 1 hour and 24 hours, the relative density of p-cPLA_2_/cPLA_2_ was significantly higher than in untreated neurons (P < 0.05 and P < 0.01 respectively) (Figure 3A). In contrast, relative expression levels of p-cPLA_2_ in neurons treated with 1 μM PACOCF_3_ and samples pre-treated with PACOCF_3_ prior to the addition of peptide for 1 hour were not significantly different from control samples. However after 24 hours, cPLA_2_ activation was reduced markedly in samples pre-treated with PACOCF_3_ in comparison to neurons treated with peptide alone (P < 0.01).

Localisation of p-cPLA_2_ in the presence of PACOCF_3_ was subsequently determined (Figure 3B). Incubation with HuPrP106-126 for 30 minutes resulted in increased p-cPLA_2_ labelling in the neurites (P < 0.01) (left panel middle row) as seen previously. In contrast, neurons pre-treated with PACOCF_3_ showed a distinct decrease in levels of p-cPLA_2_ to levels and location comparable to that of untreated controls (left panel, bottom row). The increase in p-cPLA_2_ labelling in neurons exposed to HuPrP106-126 compared to untreated neurons was still apparent after 1 hour (P < 0.01) and 24 hours (P < 0.05) (middle and right panel). In agreement with Western immunoblot data, cells pre-treated with PACOCF_3_ showed reduced levels of cPLA_2_ phosphorylation in comparison to those induced by HuPrP106-126 at all time points measured (P < 0.001, P < 0.01).

**Figure 4:**
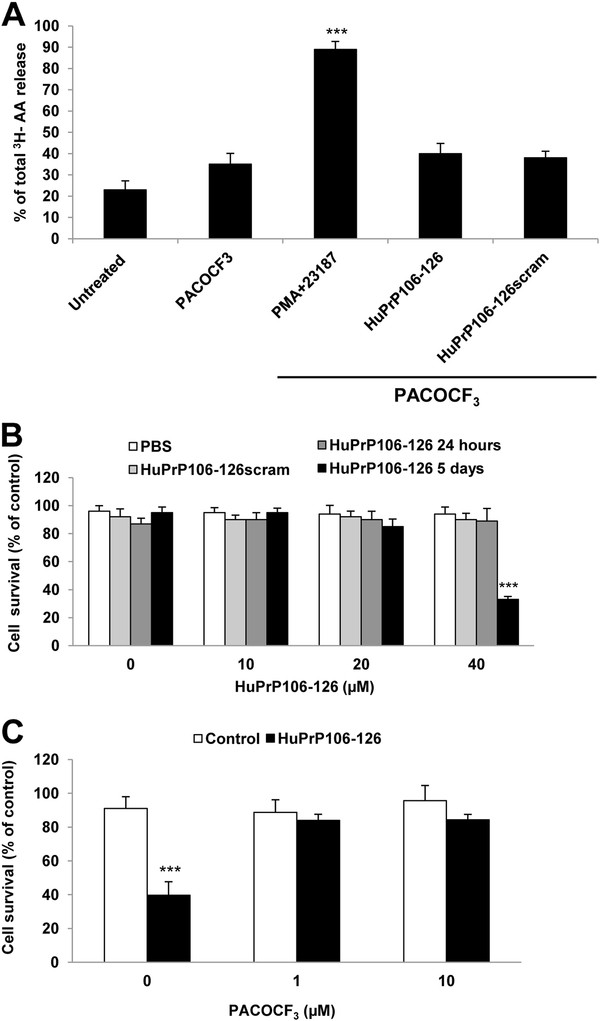
**PACOCF**_**3 **_**decreases HuPrP106-126-induced [**^**3 **^**H]-AA release and cell death in primary cortical neurons.** (**A**) Primary cortical neurons were labelled with [^3^ H]-AA for 24 hours, and subsequently pre-treated for 3 hours with 1 μM PACOCF_3_ before adding peptides or 1 μM PMA + 5 μM A23187. Cells were lysed after 24 hours and levels of [^3^ H]-AA measured in supernatant and lysates to calculate a% [^3^ H]-AA release, ***P < 0.001. (**B**) Primary cortical neurons were incubated with HuPrP106-126 at varying concentrations for 24 hours (grey bars) or 5 days (black bars). Cell viability was measured using the MTT assay. Data expressed as mean ± S.D. of three observations taken from three experiments (n = 9), ***P < 0.001. (**C**) Primary cortical neurons were pre-treated for 3 hours with 1 μM PACOCF_3_ and subsequently exposed to 40 μM HuPrP106-126 for a further 5 days. Cell viability was measured using MTT. Data expressed as mean ± S.D. of three observations from three experiments (n = 9), ***P < 0.001.

The effect of PACOCF_3_ on [^3^ H]-AA release was examined to determine if it would prevent cPLA_2_ from accessing its substrate and inhibit HuPrP106-126-induced activation of cPLA_2_. Cells exposed to PACOCF_3_ alone released similar amounts of AA to untreated controls (Figure 4A). Again primary cortical neurons exposed to 40 μM HuPrP106-126 for 24 hours released a significant (P < 0.001) amount of [^3^ H]-AA as seen previously in Figure 2B. However, when neurons were pre-treated with PACOCF_3_ and then exposed to PrP peptide for 24 hours, only 35% of total [^3^ H]-AA was released (Figure 4A). Interestingly, PACOCF_3_ pre-treatment did not affect [^3^ H]-AA release induced by PMA or A23187.

To assess the extent of the role of cPLA_2_ in PrP peptide action the effect of PACOCF_3_ on HuPrP106-126-induced cell toxicity was also analysed. The toxicity of HuPrP106-126 was measured via MTT assay and confirmed that HuPrP106-126 was lethal to primary cortical neurons, but only after a period of around 5 days (Figure 4B). This was comparable to other studies using cortical neurons [28]. Concentrations of 10 μM and 20 μM HuPrP106-126 did not significantly affect cell survival but 40 μM HuPrP106-126 caused a 70% decrease in neuronal survival (P < 0.001). HuPrP106-126 scrambled peptide had no effect on cell viability. Pre-treatment with PACOCF_3_, was protective cells pre-treated with 1 μM or 10 μM PACOCF_3_ prior to HuPrP106-126 treatment decreased the amount of cell death to a 16% and 15% loss respectively (Figure 4C).

**Figure 5:**
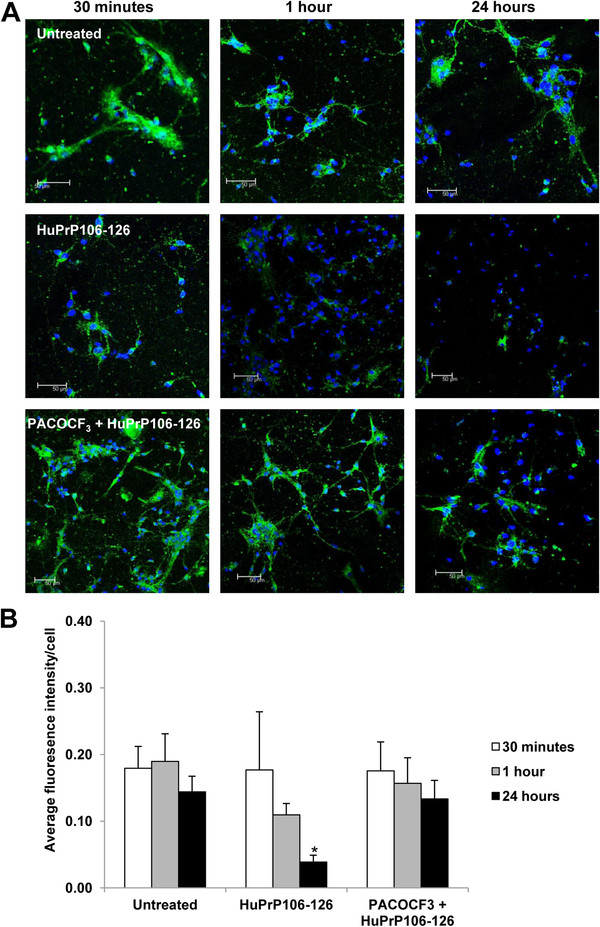
**PACOCF**_**3 **_**inhibits the decrease in synaptophysin expression caused by HuPrP106-126 treatment.** (**A**) Primary cortical neurons were either left untreated, or treated with 40 μM HuPrP106-126 for 30 minutes, 1 hour or 24 hours. Some samples were pre-treated with 1 μM PACOCF_3_ before addition of peptide. The cellular localization of synaptophysin was analyzed by immunofluorescence using anti-synaptophysin (green) antibody. The nuclei were stained with DAPI. Scale bar: 50 μM. (**B**) Intensity of synaptophysin labelling was measured and normalised to cell number. Data expressed as mean ± S.D. of three experiments, *P < 0.05.

Further investigation of the neurodegenerative effects of HuPrP106-126 was investigated by staining neurones for the synapse-related protein synaptophysin. Synapse loss is one of the signs of pathology seen in prion infection [29,30] and Alzheimer’s disease [31]. Synaptophysin can therefore be used as a neurodegenerative marker for these disorders. Cortical neurons exposed to HuPrP106-126 for 30 minutes and then examined by confocal microscopy showed no significant changes in levels of synaptophysin (Figure 5A left panel). However, longer incubations of neurones with HuPrP106-126 peptide resulted in a significant loss of synaptophysin labelling at 1 and 24 hours (P < 0.05) (Figure 5A middle and right panel). PACOCF_3,_ in addition to inhibiting HuPrP106-126-induced cPLA_2_ activation and AA-release, also protected against synapse damage with synaptophysin levels in neurons pre-treated with PACOCF_3_ prior to peptide treatment being equivalent to untreated controls (Figure 5 bottom row).

### p-cPLA_2_ co-localises with beta III tubulin in neurons exposed to HuPrP106-126

**Figure 6:**
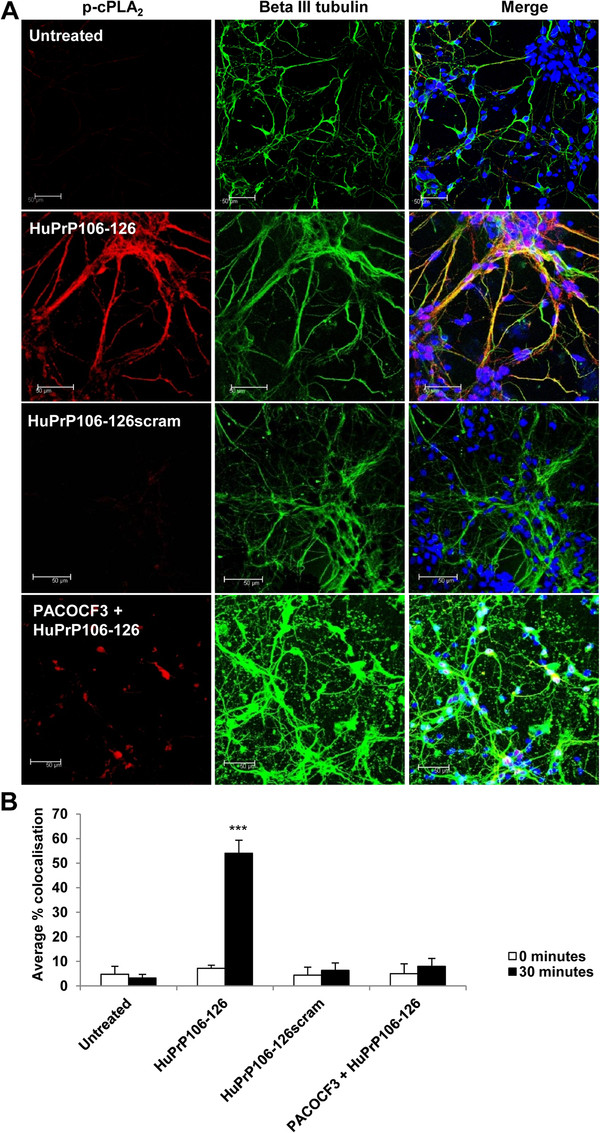
**p-cPLA**_**2 **_**and beta III tubulin colocalise in neurons after HuPrP106-126 treatment and is inhibited by PACOCF**_**3**_**.** (**A**) Primary cortical neurons were untreated, treated with 40 μM HuPrP106-126, HuPrP106-126scram, or pre-treated with 1 μM PACOCF_3_ prior to addition of peptide. p-cPLA_2_ and beta III tubulin was analyzed using anti-p-cPLA_2_ (red) and anti-beta III tubulin (green) antibodies. Nuclei were stained with DAPI. Images show a representative picture of three individual experiments. Scale bar: 50 μM (**B**) Colocalisation of the two antibodies was measured by correlation. An overall average colocalisation of all results is shown. ***P < 0.001.

**Figure 7:**
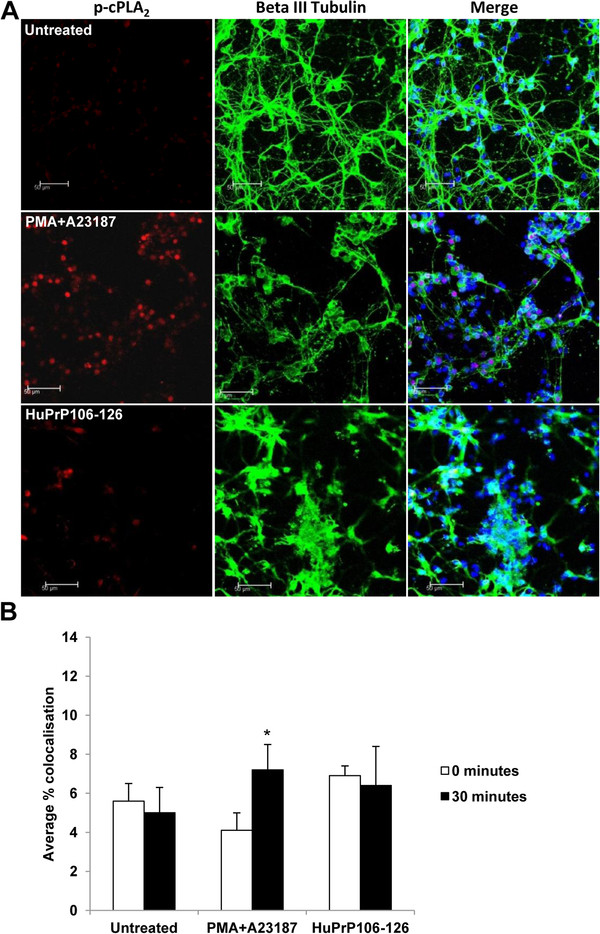
**p-cPLA**_**2 **_**and beta III tubulin do not colocalise in PrP null neurons treated with HuPrP106-126.** (**A**) Primary cortical neurons from PrP null mice were left untreated, or treated with 1 μM PMA + 5 μM A23187, or 40 μM HuPrP106-126 for 30 minutes. Images show a representative picture of two individual experiments. Scale bar: 50 μM. (**B**) Colocalisation of the two antibodies was measured by correlation. An overall average colocalisation of all results is shown. *P < 0.05.

As the translocation of p-cPLA_2_ within neuronal neurites in the presence of HuPrP106-126 had not been previously reported the question whether HuPrP106-126 treatment leads to a potential re-arrangement of the cytoskeleton, as described similarly for PrP^c^[32] was investigated. To examine the process by which HuPrP106-126 induced p-cPLA_2_ translocation, neurons were labelled with a range of cytoskeletal markers, including actin, vimentin and beta III tubulin. p-cPLA_2_ did not colocalise with actin or vimentin prior to or after treatment with HuPrP106-126 (data not shown). However, 4.8% of p-cPLA_2_ colocalised with beta III tubulin before peptide treatment, and this increased to 54% (P < 0.001) after 30 minutes exposure to HuPrP106-126 (Figure 6A and B). Colocalisation was predominantly in the neurites of the cells. Cells pre-treated with PACOCF_3_ before exposure to peptide had low levels of cPLA_2_ phosphorylation and the average colocalisation of p-cPLA_2_ with beta III tubulin was 8%. In addition, colocalisation appeared to be in the nuclear region rather than in the neurites. The relationship between p-cPLA_2_ and beta III tubulin also appeared to be PrP-specific. 6.3% colocalisation was measured between p-cPLA_2_ and beta III tubulin after exposure to a non-toxic scrambled HuPrP106-126 peptide control (Figure 6) and 6.9% colocalisation was noted in PrP null neurons treated with HuPrP106-126 (Figure 7) both comparable to untreated control levels. Interestingly p-cPLA_2_ labelling in PrP null neurons was localised to the nucleus after HuPrP106-126 exposure, indicating that PrP could define the localisation of p-cPLA_2_ within the cell.

## Discussion

It has previously been shown that PLA_2_ could play a key role in the neurodegenerative processes in prion disease pathogenesis [11-13]. The aim of the present study was to examine the effects of neurotoxic PrP peptide on cPLA_2_ activation and location in murine primary cortical neurons. Exposure of primary cortical neurons to HuPrP106-126 significantly increased levels of p-cPLA_2_ with subsequent release of AA prior to synapse loss and subsequent cell death. It was also shown that cPLA_2_ translocated to a novel region of the neuron in a PrP-dependent manner when stimulated by HuPrP106-126 this was not seen when stimulated by other PLA_2_ agonists PMA and A21387 suggesting that this abnormal translocation was PrP-disease related. The effects of HuPrP106-126 were prevented by an inhibitor of cPLA_2_.

The regulation of cPLA_2_ is complex and involves a variety of cellular processes including phosphorylation of serine residues and an increase in intracellular calcium to induce membrane translocation [20,23]. It is not clear how cPLA_2_ translocates although shuttle proteins and attraction to lipids or proteins within the membranes have been suggested [33].

Here confocal microscopy showed an increase in p-cPLA_2_ after 30 minutes incubation with HuPrP106-126 but without apparent translocation to the ER or nuclear membrane as described in previous reports [34,35] and as found using PMA and A23187 in this study. One explanation for this could be that PrP peptides may be interacting with intracellular PrP rather than glycosylphosphatidylinositol (GPI)-linked PrP at the cell surface and this may promote p-cPLA_2_ to localise in neurites. PrP^C^ is usually anchored to the cell membrane via a GPI anchor [36], however transmembrane forms of PrP, ^Ctm^PrP and ^Ntm^PrP exist that are localised to the endoplasmic reticulum membrane [37]. Indeed, the peptides used in this study contain part of the transmembrane ^Ctm^PrP region [38]. Alternatively, the HuPrP106-126 may interact with PrP^C^ to cause clustering of PrP-GPIs [39] and create altered intracellular signalling resulting in p-cPLA_2_ locating to neurites.

In this study colocalisation of p-cPLA_2_ and beta III tubulin after exposure to HuPrP(106–126) was demonstrated. As HuPrP106-126 has been shown to bind directly to tubulin [40], the data presented here potentially indicate a direct interaction of HuPrP106-126 and p-cPLA_2_ involved beta III tubulin filaments. In line with that observation, PrP^C^ has been shown to associate with tubulin in porcine and Syrian hamster brain extracts [41-43]. This is in contrast to a previous study [44], which concluded that cPLA_2_ co-immunopreciptated with actin.

Interaction of PrP peptide and p-cPLA_2_ with tubulin could facilitate their transport to neurite and axon terminals to mediate the disease-associated synapse degeneration as axonal membrane proteins are transported via microtubules [45]. The interaction of PrP with tubulin has been related to the active transport of PrP in cells, by anterograde and retrograde movement via a kinesin/dyenin mechanism in N2a cells [46], in hamster peripheral nerves [47] and in the entorhinal cortex of rats [48]. Therefore, any association of cPLA_2_ with tubulin could bring it into close association with PrP. Indeed, p-cPLA_2_ co-immunoprecipitated with PrP^Sc^ in ScGT1 cells [49] and cPLA_2_ colocalises transiently with the murine PrP peptide MoPrP(105–132) in untreated neuroblastoma cells [38]. This supports the hypothesis that there is a direct interaction of cPLA_2_ with tubulin in the presence of HuPrP106-126 and endogenous PrP_._^C^ Any subsequent disruption of the microtubules could lead to induction of apoptosis [50].

The present study also supports previous reports that activation of cPLA_2_ is associated with PrP peptide-induced neurotoxicity [51]. Indeed the PLA_2_ inhibitor PACOCF_3_ inhibited cPLA_2_ phosphorylation in primary cortical neurons even after 24 hours of HuPrP106-126 exposure. Furthermore, only low levels of p-cPLA_2_ were seen by confocal microscopy and colocalisation with beta III tubulin was lost. This correlated with prevention of ^3^ H]-AA release and ultimately cells were protected against synaptic synaptophysin loss and neuronal death. This is consistent with published data where levels of synaptophysin have been found to decrease in brains of CJD patients [52] and murine scrapie models [53]. The finding that PACOCF_3_ did not appear to affect ^3^ H]-AA release induced by PMA and A23187 are consistent with a report that PMA induced activation of cPLA_2_ may not involve the Ser505 phosphorylation site therefore the mechanism of PACOCF_3_ may not effect PMA and A23187-induced cPLA_2_ phosphorylation [54]. Alternatively the agonist A23187 has been shown to have membrane perturbing effects leading to non labelled fatty acid release, therefore the lack of effect of PACOCF_3_ on ^3^ H]-AA release induced by PMA and A23187 could be due to a high amount of unlabelled material in the culture medium [55]. PACOCF_3_ pre-treatment inhibited the loss of synaptophysin, indicating that PLA_2_ could be implicated in synapse degeneration and loss of synaptophysin. It also suggests that PLA_2_ changes occur as an early event prior to neuronal death, again consistent with *in vivo* data [11,56] in which prostaglandin E_2_, an end-product of the PLA_2_ pathway was elevated in specific brain regions before the onset of detectable neuronal loss. Activated microglia also precedes neuronal loss and is a pathological hallmark of prion disease [56]. The neurotoxicity of PrP peptides are greatly enhanced in the presence of microglia [3], however microglial cell contamination of neuronal cultures as measured by GFAP labelling was negligible in this study therefore this is unlikely to have affected results.

The implication that PLA_2_ changes are a preliminary occurrence prior to cell death is also compounded by the toxicity data seen in this study cortical neurons show a significant decrease in metabolic activity after 5 days whereas an increase in p-cPLA_2_ is seen after 30 minutes. It is likely that the increase in cPLA_2_ phosphorylation encourage the cell into a cascade of intracellular signalling events which eventually lead to cell death. This is the first report of PACOCF_3_ protecting neurons against PrP-induced toxicity however the PACOCF_3_ analogue arachidonyl trifluoromethyl ketone (AACOCF_3_) has previously been reported to reduce PrP^Sc^ levels in neuroblastoma cells [13]. The results presented here are also consistent with earlier data showing an increased survival of SHSY-5Y cells treated with the PACOCF_3_ analogue AACOCF_3_ before exposure to HuPrP106-126 [22].

Thus cPLA_2_ inhibition by AACOCF_3_ and PACOCF_3_ indicates a vital role for cPLA_2_ activation in neuronal death, and indicates a potential role for the use of cPLA_2_ inhibitors for neuroprotection in neurodegenerative diseases. PACOCF_3_ and AACOCF_3_ are both trifloromethyl ketone analogues of fatty acids [57]; AACOCF_3_ inhibits cPLA_2_ by binding to its active site [58] and it is likely that its analogue PACOCF_3_ acts in a similar way. As PLA_2_-induced AA release causes free oxygen radical release membrane disruption [14] and subsequent cellular damage, the neuroprotective action of PACOCF_3_ and other PLA_2_ inhibitors could be through inhibition of these events, and/or through disrupting colocalisation of PrP^Sc^, p-cPLA_2_ and beta III tubulin, by attaching to the active site of cPLA_2_. Whether cPLA_2_ is activated via direct cell exposure to neurodegenerative peptides or is due to a secondary event remains to be investigated.

## Conclusions

We provide evidence that neurotoxicity resulting from exposure to prion peptide HuPrP106-126 is dependent on cPLA_2_ phosphorylation and a novel translocation to neurites coupled with an interaction with beta III tubulin. Inhibition of cPLA_2_ activation provides protection to neurons from PrP peptide induced synapse damage and cell death. Taken together these findings suggest a vital role for cPLA_2_ in the progress of neurodegeneration caused by prion peptides.

## Methods

### Reagents and antibodies

PMA A23187, poly-D-lysine, paraformaldehyde, Tris–HCl, SDS, glycerol, Na_3_VO_4_ and palmitoyl trifluoromethyl ketone (PACOCF_3_) were obtained from Sigma (Dorset, UK). Primary antibodies included mouse monoclonal cPLA_2_ (4-4B-3C, sc-454), rabbit polyclonal anti-phosphorylated cPLA_2_ (Ser505 residue, sc-34391), goat polyclonal anti-synaptophysin (sc-7568) (Santa Cruz Biotechnology, Santa Cruz, CA) and goat polyclonal beta III tubulin (Abcam, Cambridge). Fluorescently conjugated secondary antibodies included anti-rabbit Alexa Fluor555 and anti-goat Alexa Fluor 488 (Invitrogen, Paisley, UK).

### Isolation of primary cortical neurons

Primary cortical neurons were prepared from day 15.5 gestation CD-1 mice (Charles River Margate, UK), or 129/Ola PrP null mice originally obtained from Jean Manson as previously described [59]. Primary cortical neurons were prepared from the brains of mouse embryos as described previously [60] and seeded into 24-well or 96-well trays and maintained in neurobasal media (NBM) with B27 components (Invitrogen, Paisley, UK), for 7 days prior to use.

### Peptide-induced cell death assays

Primary cortical neurons were seeded at a density of 1x10^6^ cells per ml in NBM and left in culture for 7 days. Cells were subsequently treated for 5 days with 10 μM – 40 μM HuPrP106-126 or HuPrP106-126 scrambled (Bachem St. Helens, UK)*.* To ensure homogenous distribution in solution, peptides were sonicated 3 x 10 seconds prior to use. In inhibitory experiments, neurons were pre-treated with 1 μM or 10 μM of the PLA_2_ inhibitor PACOCF_3_ for 3 hours before the addition of peptide or vehicle control. After incubation, medium was removed and cell survival was measured by the addition of 500 μg ml^-1^ of 3,[4,5 dimethylthiazol-2yl]-2,5 diphenyltetrazolium bromide (MTT) (Sigma) which is reduced to a formazan dye in metabolically active cells. MTT was left on the cells for 3 hours with the culture tray kept at 37°C. Cells were then lysed and the MTT-formazan salt was solubilised by the addition of 100 μl per well of DMSO. Optical density was quantified using a SpectraMax M2 microplate reader (Molecular Devices, CA, USA) at a wavelength of 550 nm. Absorbance measured in MTT assays was expressed as percent of the untreated controls (defined as 100%).

### Measurement of [^3^ H]-arachidonic acid release

Primary cortical neurons were seeded at 2 x 10^5^ cells per well in 24-well trays and left in culture for 7 days. Cells were then incubated for 24 hours with 1 μCi per ml (specific activity 212 Ci per mmol) [5,6,8,9,11,12,14,15-^3^ H]AA (Amersham Biosciences Amersham, UK) in NBM. Cells were washed twice in NBM and new media containing 0.3% fatty acid free BSA (PAA, Somerset, UK) and 40 μM HuPrP106-126 or scrambled peptide was added. Positive controls included a PKC activator, phorbol 12-myristate 13-acetate (PMA) used at 1 μM, with 5 μM of the calcium ionophore, A23187. In some experiments 1 μM PACOCF_3_ was added in media for 3 hours before the addition of peptides. Supernatants were taken after 0, 15 or 30 minutes or 24 hours and centrifuged for 5 min at 16000 x *g*. Cells were lysed in lysis buffer (63.5 mM Tris–HCl (pH 6.8), 10% (v/v) glycerol, 2% (w/v) SDS, 1 mM Na_3_VO_4_, HALT protease inhibitor cocktail (Pierce, Northumberland, UK). Samples were added to 4 ml scintillant and radioactivity was determined by β-scintillation counting (Packard Tri Carb Liquid Scintillation Analyser, Counter B 1990).

### Confocal microscopy

Murine cortical neurons were seeded onto poly-D-lysine coated coverslips at 2 x 10^5^ cells per coverslip and left in culture for 7 days. Cells were treated with 40 μM HuPrP106-126 or a scrambled peptide control. In some experiments 1 μM PACOCF_3_ was added in NBM for 3 hours before the addition of peptides. The media was removed and cells washed in PBS before being fixed with 4% paraformaldehyde (PFA) pH 7.4 for 15 min at room temperature. Cells were subsequently permeabilised in 0.1% (v/v) Triton-X100 (Sigma Dorset, UK) in PBS for 10 min at room temperature. Cells were washed, and then incubated with 1% (v/v) donkey or rabbit serum (Chemicon) in PBS for 1 hour at room temperature to block. Cells were then exposed to primary antibody in diluent (1% donkey or rabbit serum) in PBS overnight at 4°C. The coverslips were then washed and counterstained with the appropriate AlexaFluor secondary antibody for 1 hour. Coverslips were washed again, incubated with phalloidin (5 U per ml) for 20 min and exposed to 4 μg per ml DAPI in PBS for 10 min at room temperature to label nuclei. Cells were given a final rinse with ultrapure water before being mounted onto Superfrost Plus slides using Vectashield (Vector Labs, Peterborough, UK) and sealed with Eukitt (Agar Scientific, Stansted, UK). Fluorescence was visualised using a Leica SP5 RS confocal microscope (Leica Microsystems, Milton Keynes, UK) with HC PL Fluotar 20x 0.5 dry objective.

### Western blotting

Primary cortical neurons were seeded at 5 x 10^5^ cells per well in 24 well-plates and left in culture for 7 days respectively. NBM was removed and replaced with media containing 40 μM HuPrP106-126. Some cells were pretreated with 1 μM PACOCF_3_ prior to addition of peptide. After removal of supernatant cells were washed with ice-cold PBS and 0.4 mM sodium orthovanadate (Na_3_VO_4_) (Sigma). Cells were lysed in 100 μl of ice-cold lysis buffer (63.5 mM Tris–HCl (pH 6.8), 10% (v/v) glycerol, 2% (w/v) SDS, 1 mM Na_3_VO_4_, HALT protease inhibitor cocktail (Pierce, Northumberland)). 20 μg per lane of protein was resolved using a graduated 4-12% Novex Tris-glycine gel (Invitrogen) using the XCell Surelock Mini-Cell apparatus (Invitrogen). Gels were run at 125 V for around 1.5 hours (Bio-Rad, Hemel Hempstead, UK). Proteins were transferred onto nitrocellulose membrane (Amersham Biosciences, Buckinghamshire, UK) at 25 V for 60 min and then blocked for two hours at room temperature in PBS containing 5% milk powder (w/v) and 0.1% (v/v) Tween 20 (PBST) (Sigma). Membranes were incubated overnight at 4°C in PBST with 5% (w/v) milk powder containing anti-cPLA_2_ mAb (1:1000), phosphospecific anti-cPLA_2_ polyclonal (p-cPLA_2_) (1:1000), (Santa Cruz Biotechnology, Santa Cruz, CA) and anti-β-Actin mAb (1:5000) (Chemicon, Hampshire, UK). Blots were washed in PBST (1 x 15 min, 3 x 5 min) and incubated with goat anti-mouse or rabbit IgG horseradish peroxidase-conjugated antibody (1:2000) (Pierce) for two hours at room temperature. After washing, an enhanced chemiluminescence kit (Amersham Biosciences) was used to detect bound antibody. Blots were analysed using ImageJ software (Rasband, W.S., ImageJ, U. S. National Institutes of Health, Bethesda, Maryland, USA, http://rsb.info.nih.gov/ij/, 1997–2007).

### Statistical analysis

Results from at least three independent experiments are expressed as mean ± standard deviation (S.D.) unless stated otherwise. Colocalisation of molecules in confocal microscopy was calculated using Pearson’s correlation coefficient using Leica LAS AF software (Leica, Milton Keynes, UK). The intensity of the fluorescent label of interest was measured using ImageJ software (version 1.41, Maryland, USA). The overall intensity was divided by the number of cells counted in replicate experiments to give an average intensity for all images. Statistical differences between means were calculated using Student’s *t*-test or one-way analysis of variance (ANOVA) with a Bonferroni *post hoc* test using SPSS software (version 14.0, Chicago, USA), with significance set at P < 0.05.

## Abbreviations

cPLA_2_: Cytosolic phospholipase A_2_; AA: Arachidonic acid; PACOCF_3_: Palmitoyl trifluoromethyl ketone; TSEs: Transmissible spongiform encephalopathies; BSE: Bovine spongiform encephalopathy; CJD: Creutzfeldt-Jakob Disease; PrP: Prion protein; PMA: Phorbol 12-myristate 13-acetate; GPI: Glycosylphosphatidylinositol; AACOCF_3_: Arachidonyl trifluoromethyl ketone.

## Competing interests

The authors declare they have no competing interests.

## Author contributions

VL participated in the design of the study, carried out the experiments, performed the statistical analysis and drafted the manuscript. AW participated in the design of the study and helped draft the manuscript. DW participated in the design and coordination of the study and helped draft the manuscript. All authors read and approved the final manuscript.

## Supplementary Material

Additional file 1 **Figure S1.PMA and A23187 induce cPLA**_**2 **_**activation and subsequent release of arachidonic acid within 30 minutes.** Primary cortical neurons were labelled with [^3^ H]-AA for 24 hours then treated with 1 μM PMA and 5 μM A23187 for 30 minutes. Cells were lysed and levels of [^3^ H]-AA measured in supernatant and lysates to calculate a% [^3^ H]-AA release. Data expressed as mean ± S.D. of three experiments. **P < 0.01, ***P < 0.001. Click here for file
