# Supplementary figures and images for: Inhibition of cytosolic Phospholipase A2 prevents prion peptide-induced neuronal damage and co-localisation with Beta III Tubulin
Source: BMC Neurosci. 2012 Aug 28;13:106. doi: 10.1186/1471-2202-13-106 (PMC3496594; doi:10.1186/1471-2202-13-106)

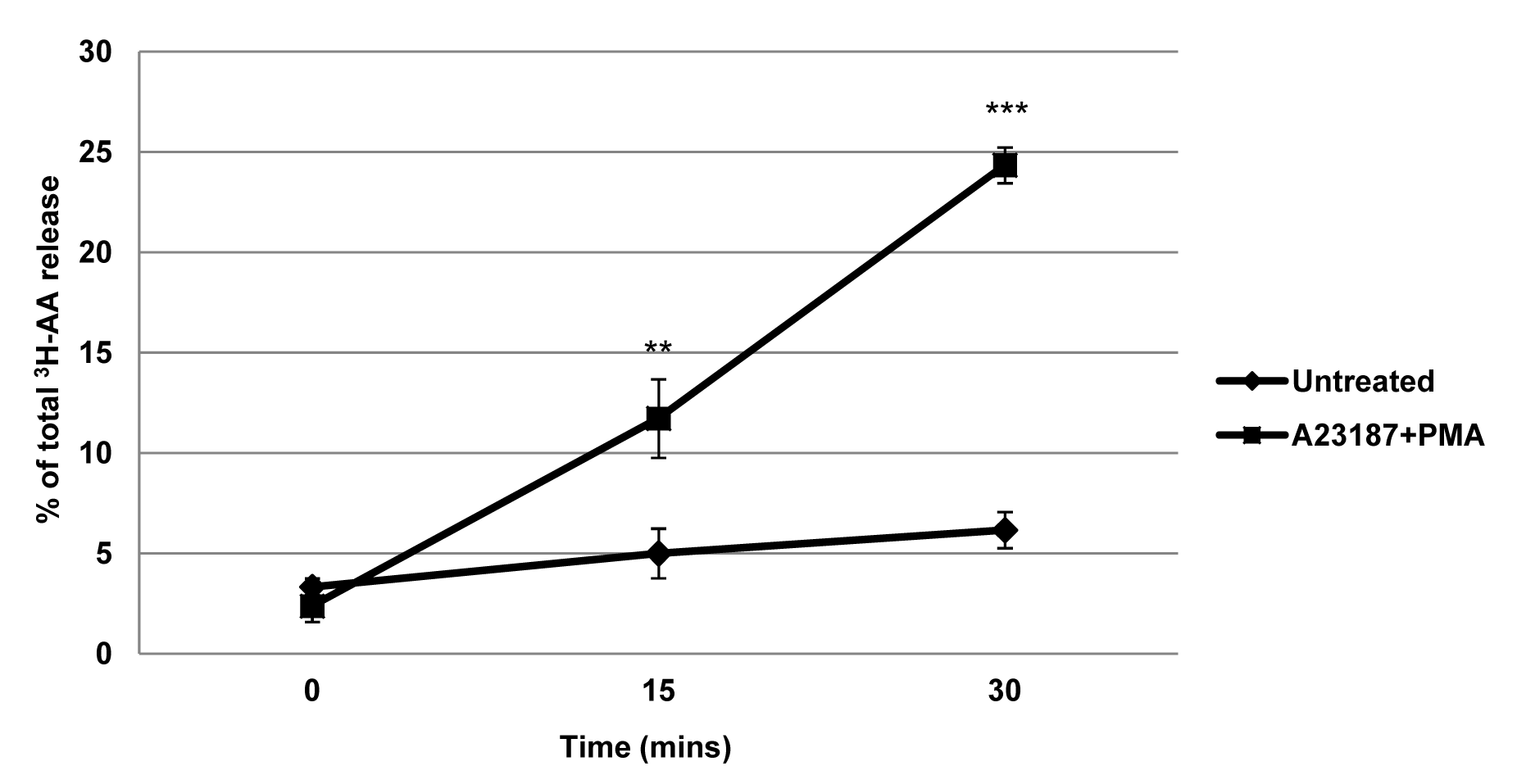

Supplement: Additional file 1 — Figure S1.PMA and A23187 induce cPLA2 activation and subsequent release of arachidonic acid within 30 minutes. Primary cortical neurons were labelled with [3 H]-AA for 24 hours then treated with 1 μM PMA and 5 μM A23187 for 30 minutes. Cells were lysed and levels of [3 H]-AA measured in supernatant and lysates to calculate a% [3 H]-AA release. Data expressed as mean ± S.D. of three experiments. **P < 0.01, ***P < 0.001. [file 1471-2202-13-106-S1.tiff]
